# The Performance Demands and Technical Determinants for Tackle and Ruck Success During the Pool and Knockout Stages of the Men's International World Rugby Sevens Series

**DOI:** 10.1002/ejsc.12269

**Published:** 2025-02-13

**Authors:** Fanie de Klerk, Ben Jones, Willie Maree, Sharief Hendricks

**Affiliations:** ^1^ Division of Physiological Sciences Department of Human Biology Faculty of Health Sciences University of Cape Town Cape Town South Africa; ^2^ Stellenbosch Football Club Stellenbosch South Africa; ^3^ Carnegie Applied Rugby Research (CARR) Centre Carnegie School of Sport Leeds Beckett University Leeds UK; ^4^ Premiership Rugby London UK; ^5^ England Performance Unit Rugby Football League Manchester UK; ^6^ School of Behavioural and Health Sciences Faculty of Health Sciences Australian Catholic University Brisbane Australia; ^7^ South African Rugby Union Cape Town South Africa

## Abstract

The purpose of this study was to compare tackle and ruck frequencies between pool and knockout matches during the Men's International World Rugby Sevens Series and also determine which technical determinants increase the likelihood of tackle success within each stage of the tournament. Video analysis of all matches during the 2018/2019 International Men's Rugby Sevens World Series was conducted (*n* = 449 matches). This equated to 21226 tackle contact events and 6345 rucks events. Each tackle event was further coded for tackle descriptors (type of tackle, direction of contact and point of body contact) and tackle outcomes (successful and unsuccessful). No differences were found between the mean tackles per match of pool and knockout stages (pool 47.5, 95% CI 46.5–48.6 vs. knockout 46.9, 95% CI 45.7–48.0). There was a significant difference (*p* < 0.001) in mean rucks per match between pool and knockout stages (pool 14.8, 95% CI 14.2–15.4 vs. knockout 13.3, 95% CI 12.7–13.9). In conclusion, tackle frequencies per match remained consistent across the series and between the different competition stages and match halves. Ruck frequencies on the other hand decreased from the first tournament to latter parts of the series, and fewer rucks were observed in the knockout stage of the tournaments. The frequency and higher likelihood of tackle success for arm tackles in Sevens highlights a unique demand of Sevens, which strengthens the argument for Sevens‐specific tackle training and coaching.

**Summary**

- Tackle frequencies per match remained consistent across the series and between the different competition stages and match halves, whereas ruck frequencies decreased from the first tournament to latter parts of the series, and fewer rucks were observed in the knockout stage of the tournaments.
- In both the pool and knockout stages, the most frequently occurring type of tackle was the arm tackle. In the pool stages, the arm and jersey tackle were also associated with a higher likelihood of tackle success compared to other tackle types.
- The findings of this study highlight the unique technical‐tactical tackle and ruck demands of Sevens, which strengthens the argument for Sevens specific tackle and ruck training and coaching.

## 1 Introduction

Rugby Sevens (henceforth called ‘Sevens’) is an Olympic sport that has grown rapidly worldwide, both in terms of participation and commercialisation (World Rugby 2021). Like other rugby football codes (rugby union and rugby league), Sevens is characterised by frequent dynamic physical–technical contests interspersed between intermittent high‐intensity running (Ross, Gill, and Cronin 2015; Higham et al., 2012 2013; Meir 2012; Hughes and Jones 2005). The most frequent of these physical–technical contests are the tackle and ruck (Ross, Gill, and Cronin 2015; Hendricks et al. 2020; Paul et al. 2022). The tackle contest is observed when the defending player(s), known as the tackler(s), attempts to impede the attacker's (the ball‐carrier) progression towards the try‐line and regain possession of the ball through an action known as tackling (Quarrie and Hopkins 2008; Hendricks and Lambert 2010; Hendricks et al. 2020a). The ruck contest typically occurs after the tackle and occurs when opposing players are in contact and on their feet contesting the ball on the ground (Hendricks et al. 2018 2020, 2020a). A meta‐analyses of video analysis studies in Sevens report that on average, 14 (95% CI 0–33) tackles and 5 (95% CI 0–12) rucks occur during a 14‐min Sevens match (Paul et al. 2022). Considering the frequency of the tackle and ruck, and that each of these contests is an opportunity to (re)gain possession of the ball and prevent a try being scored, it is no surprise that success in Sevens is, in part, dependent on the teams' ability to repeatedly ‘win’ these physical–technical contests (Barkell, O’Connor, and Cotton, 2017b; Higham et al., 2014 2014a; Hendricks et al. 2020). The ability to successfully contest the tackle is also a key player performance indicator and a strategy to reduce injury risk in matches (Hendricks and Lambert 2010; Hendricks et al. 2018a; Behardien et al. 2024)—as such, improving player safety and performance in the tackle is the top priority for rugby stakeholders and governing bodies (Hendricks et al., 2023).

In addition to the Olympics, World Rugby (the sports governing body) has run a World Sevens Series since 1999, featuring national teams from all rugby nations. The series grew to 10 tournaments in 10 different countries, across five continents, and played over a period of seven months. The series is played annually, with points collected based on a team's performance at each tournament. At the end of the series, the team with the most points is crowned world champions (World Rugby 2021a). More recently, the series has undergone a change with seven tournaments taking place in a regular season and a grand final hosting the top eight teams (World Rugby 2024). The series within the Olympic year also acts as qualifiers for the Games, with the top 4 teams automatically qualifying (World Rugby 2024a). For both the series and the Olympics, the tournament structure uses a pool stage (four teams in each pool) to a knockout stage format, whereby teams will play against all opponents in their respective pools with the top two teams from each pool progressing through the knockout stages. Studies in rugby union and Sevens have shown that match variables that may predict match outcomes differ between pool and knockout stages (Bennett et al. 2021; Barkell, O’Connor, and Cotton 2017a) and that knockout stages are more evenly matched (Barkell, O’Connor, and Cotton 2017a). In line with these studies and considering that all major Sevens tournaments use a pool to the knockout stage format, understanding the tackle and ruck demands within and between the competition stages will help coaches better prepare for the technical and tactical demands of each stage—both in training and during the transition period between the pool and knockout stages.

Studying the frequency of occurrence of match variables is considered ‘what’ studies since they describe key events over a match, for different stages of a tournament or between match periods (e.g., 1^st^ half vs. 2^nd^ half) (Den Hollander et al. 2018). In team sports, conducting ‘what’ studies is the first step in developing effective training programmes as they identify key events and key competition periods while also gaining an overall sense of the demands of the game (Den Hollander et al. 2018). Subsequent to identifying key events and key competition periods, the next step in developing effective training programmes is to study which factors increase the likelihood of success for these key events—these are considered ‘how’ studies, since they typically describe how certain factors relate to an outcome (performance or injury). In Sevens, only two studies have attempted to describe how certain factors relate to a successful performance outcome. Barkell, O’Connor and Cotton (2018) identified the actions required for winning the ruck contest, whereas Hendricks et al. (2020) identified which technical determinants are associated with tackle and ruck performance outcomes. For example, as a tackler, smother tackles and strong leg driving post‐contact prevented tackle breaks and reduced the likelihood of the attacking team maintaining possession (Hendricks et al. 2020). Both of these studies use a sample of matches from the Sevens Series and do not differentiate between pool and knockout stages. In view of all the above, the purpose of this study was to describe tackle and ruck frequencies across an entire World Sevens Series and specifically compare competition stages (pool vs. knockout stages) and match periods (1^st^ half vs. 2^nd^ half). A secondary aim was to identify tackle determinants associated with tackle performance.

## 2 Methods

All matches from the 2018/2019 International Men's Rugby Sevens World Series were analysed for this study. This annual series includes 10 tournaments spread throughout the year. The total number of matches equated to 449 matches (*n* = 449) over 10 tournaments (45 matches per tournament). One match (Tournament 1, match 9) was excluded from the analyses due to the incomplete video footage. The 16 teams (15 core teams and 1 invitational team per tournament) played 5–6 games over 2–3 days per tournament. This equated to 21226 tackle contact events and 6345 rucks events. Two knockout matches finished in a draw after the conclusion of ‘normal’ time. These matches progressed to extra time with ‘sudden death’ rules applied—the first team to score points wins the match and progresses to the next stage of the tournament. Tackle and ruck events from extra time were excluded due to the potential skewing of data. The study was approved by the University Research Ethics Committee (HREC REF: 625/2021).

The video footage of all matches were analysed using SportsCode Elite version 6.5.1, using an Apple MacBook Pro (Apple Inc., Cupertino, California USA). The software enabled the analyst to control the time lapsed, as well as record and save each coded instance into an electronic database. The analyst was also able to pause, rewind and watch the footage in slow motion. The highest frame frequency, the match footage could be slowed down to, was 25 frames per second. Match instances were coded using variables and definitions described in previous studies (Hendricks et al. 2014 2018, 2020 2020a) (Table 1). A tackle event was defined as ‘any event where one or more defenders made contact with the ball‐carrier regardless of whether the player went to ground or not’ (Hendricks et al. 2014 2018, 2020 2020a; Hendricks and Lambert 2010; Quarrie and Hopkins 2008). If the tackle contest led to a ruck, the ruck event was also coded. A ruck was defined ‘as any event where one or more players from opposing teams made contact around the ball while keeping on their feet’ (Hendricks et al. 2018 2020, 2020a). For this study, all tackle events were coded for tackle contact determinants and outcomes (Table 1). Tackle contact determinants were coded when contact was first made between the tackler and ball‐carrier. The tackle contact determinant variables coded at this point were the type of tackle, tackle direction, point of contact on the ball‐carrier, tackle sequence and number of tacklers. Thereafter, the outcome of the tackle (successful/unsuccessful) was coded for. If the outcome of the tackle led to a ruck, the ruck event was also recorded. All video footages were provided by the South African Rugby Union.

**TABLE 1:** Tackle variables and definitions.

Variables	Definition
*Defender*	Player/s involved in the tackle on the defending team
1. Total tackles	Number of tackle contact events
2. Type of tackle	*Arm tackle* = tackler impedes ball‐carrier with upper limbs
	*Collision tackle* = tackler impedes ball‐carrier without the use of arms
	*Jersey tackle* = tackler holds ball‐carrier's Jersey
	*Lift tackle* = tackler raises ball‐carrier's hips above ball‐carriers head
	*Shoulder tackle* = tackler contacts the ball‐carrier with the shoulder as the first point of contact
	*Smother tackle* = tackler uses chest and wraps both arms around ball‐carrier
	*Tap tackle* = tackler trips ball‐carrier with hand on lower limb below the knee
3. Direction of contact	*Front* = tackler makes contact in front of the ball‐carrier
	*Side* = tackler makes contact with the ball‐carrier's side
	*Oblique* = tackler makes contact with ball‐carrier at an angle
	*Behind* = tackler makes contact with the ball‐carrier from behind
4. Point of body contact	*Legs* = area between ball‐carrier's hips and toes
	*Mid‐torso* = above the ball‐carrier's hip level to the level of the ball‐carrier's arm pit
	*Shoulder* = from the ball‐carrier's arm pit level to the shoulder level, including the arm
	*Head and neck* = above the shoulder with any connection with the head/neck during the tackle
5. Tackle sequence	*One‐on‐one* = one defender contacts one attacker
	*Sequential* = one defender contacts one attacker followed by a second defender joining the contact situation
	*Simultaneous* = two defenders contact one attacker at the same time
6. Number in tackle	Number of tacklers in the tackle event
*Attacker*	
1. Attacker intention	*Straight* = ball‐carrier ran straight at the defence
	*Sidestep* = ball‐carrier performed an evasive step initiated by either leg
	*Lateral run* = ball‐carrier performed a run from touchline to touchline
	*Diagonal run* = ball‐carrier runs at an angle, instead of straight at the attacker
*Tackle outcome (defender)*	
1. Successful	Successful after contact, the tackler prevents the ball‐carrier and ball from progressing towards his try‐line (gain‐line success) and does not concede a penalty
2. Unsuccessful	Unsuccessful when the ball‐carrier was able to offload the ball, or break an attempted tackle, or progresses towards opposition try‐line, or an infringement was committed, or when a try was scored

To reduce inconsistencies, one analyst (FdK) was used for all analyses. The analyst studied the variables and their corresponding definitions to make certain that each variable was understood. When the analyst observed behaviours that fulfiled the definitions (e.g., jersey tackle—tackler holds ball‐carrier's jersey before impeding ball‐carrier with upper limbs), the event was coded using the software. Despite using only one coder and all efforts to increase the objectivity of the methods, subjectivity is likely when using human observation to analyse the video (O'Donoghue 2009).

### 2.1 Reliability

For intra‐coder reliability, five randomly (www.random.org) selected matches were coded twice using the variables and definitions described earlier. Coding of the same matches was separated by more than one week (Hendricks et al. 2014 2018). Kappa statistics (κ ± standard error) were used to evaluate intra‐coder reliability for each of the matches (James, Taylor, and Stanley 2007; Viera and Garrett 2005). Kappa values from 0.81 to 0.99 are considered ‘excellent’, whereas values between 0.61 and 0.80 represent ‘substantial agreement’ (James, Taylor, and Stanley 2007; O'Donoghue 2009; Viera and Garrett 2005). For this study, intra‐coder reliability for the five matches was 0.95 (±0.11), which represents ‘excellent’ agreement between the repeated measures.

### 2.2 Statistical Analyses

For the count data, a one‐way analysis of variance (ANOVA) with a Bonferroni *post‐hoc* test was used to compare tackle and ruck frequencies between the tournaments. The unpaired *t*‐test was used to compare the frequency of occurrence of successful and unsuccessful tackle events between pool and knockout matches and between match halves. All tests used a two‐tailed *p*‐value with an alpha level of the significance set at *p* < 0.05. Frequencies per match were also reported for tackle type, contact direction and point of body contact. All count data were reported as mean ± 95% confidence intervals (95% CI's).

Multinomial logistical regression and likelihood ratio tests were used to identify which tackle determinants were associated with either a successful or unsuccessful tackle outcome. The tackle determinants included the type of tackle, tackle direction, point of contact on the ball‐carrier, tackle sequence and number of tacklers. To perform the analysis, these determinant variables were computed relative to a referent or base variable. For example, for the type of tackle, the base variable was an arm tackle. Thereafter, likelihood ratio tests were conducted to test the overall effect of each independent variable on the main effects model. Independent variables that had an overall significant effect (*p* < 0.05) on the main effects model were subsequently identified. A post‐hoc adjusted specific effects model was then computed with all the variables of the significant determinant included (second stage model). Separate models were conducted for pool and knockout stages of the competition. Relative risk ratios (RRR) and 95% confidence intervals (CI's) are reported for the main effects models and the determinants of the specific effects model. Significant determinants in the specific effects model are also reported, with the alpha set at *p* < 0.05.

The RRR is a ratio of the probability of the event (outcome) occurring in the observed determinant versus the nonobserved determinant. For interpreting the multinomial logistic regression, if the RRR of the variable is more than 1, the comparison determinant is more likely to occur, and if the RRR of the variable is less than 1, the base variable is more likely to occur. The magnitude of this likelihood is represented by the RRR value. Similar analyses of rugby union and Sevens performance can be found in previous studies (Hendricks et al. 2013 2014, 2018 2020; Sewry et al. 2015). The suitability and equations for logistic regression can be found in Hamilton (2012) and Huck (2012). All statistics were computed using STATA 12 (StataCorp, College Station, TX, USA).

## 3 Results

### 3.1 Tackle and Ruck Frequency per Match

**FIGURE 1:**
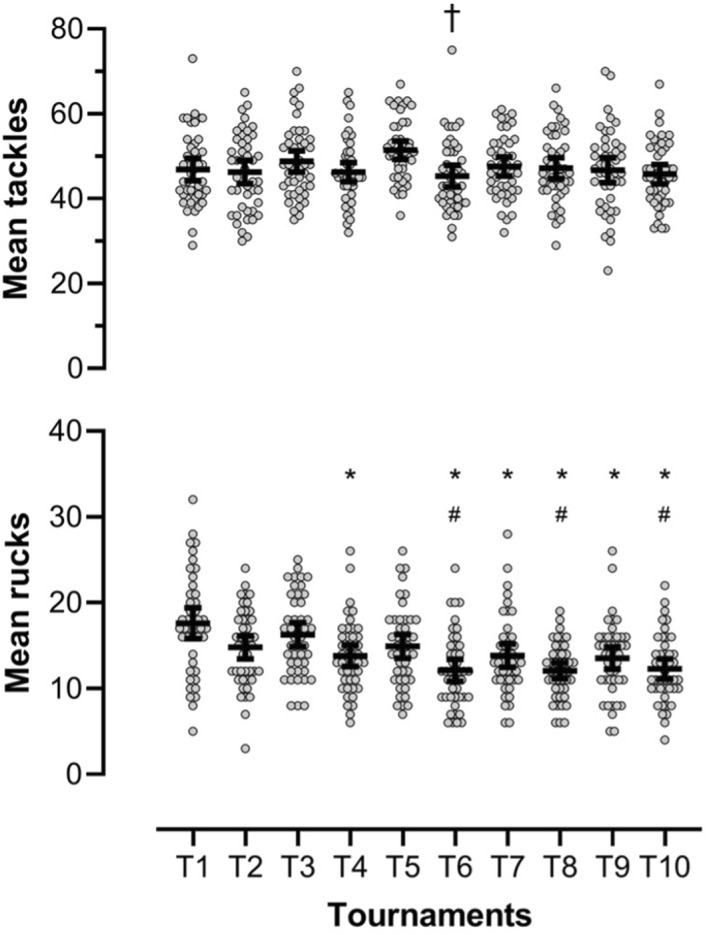
Mean ± 95% confidence intervals (95% CI's) tackle and ruck frequency across the series. † Significant differences (*p* < 0.05) in tackles compared to T5. * Sig. differences (*p* < 0.05) in rucks compared to T1. # Sig. differences (*p* < 0.05) in rucks compared to T3.

The mean tackle frequency for a match was 47.2 (95% CI 46.4–48.0) across the 10 tournaments in the 2018/2019 World Rugby Sevens Series. The mean ruck frequency for a match was 14.1 (95% CI 13.7–14.5) across the series (Figure 1).

A significant difference in tackle frequency was evident between tournaments (T) T5 and T6 (T5 51.4, 95% CI 49.3–53.5 vs. T6 45.3, 95% CI 42.8–47.7, *p* < 0.05) (Figure 1). There were no further significant differences between any other tournaments. For ruck frequency, there was a significant difference between tournament T1 and tournaments T4, T6, T7, T8, T9 and T10 (Figure 1). There were also significant differences between tournaments T3 and T6 and T8 and T10 (Figure 1).

**FIGURE 2:**
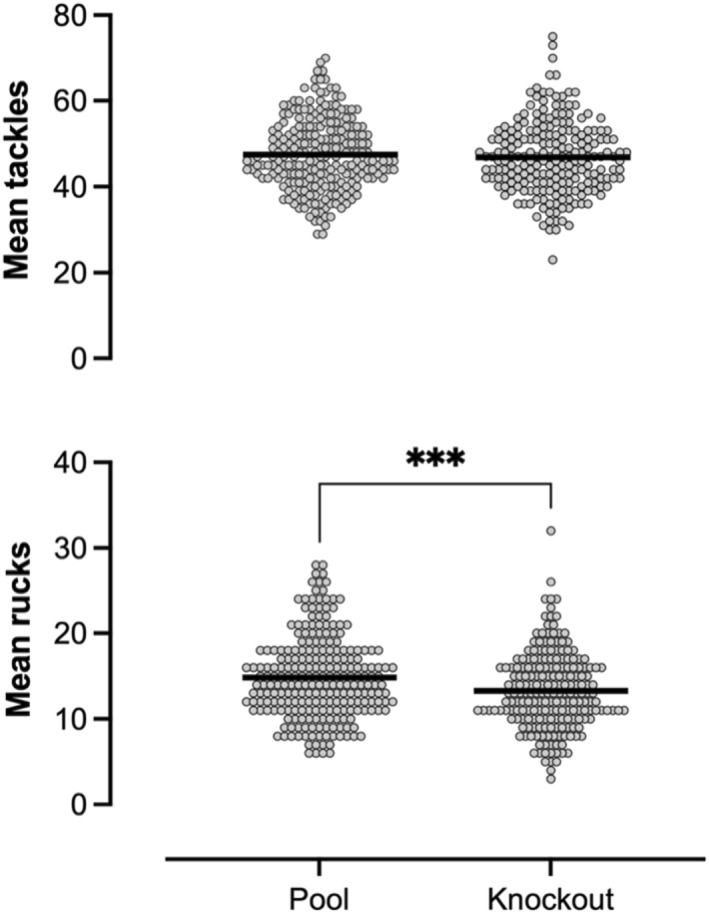
Mean tackles and rucks per match between the stages of competition. *** Sig. differences (*p* < 0.001).

**FIGURE 3:**
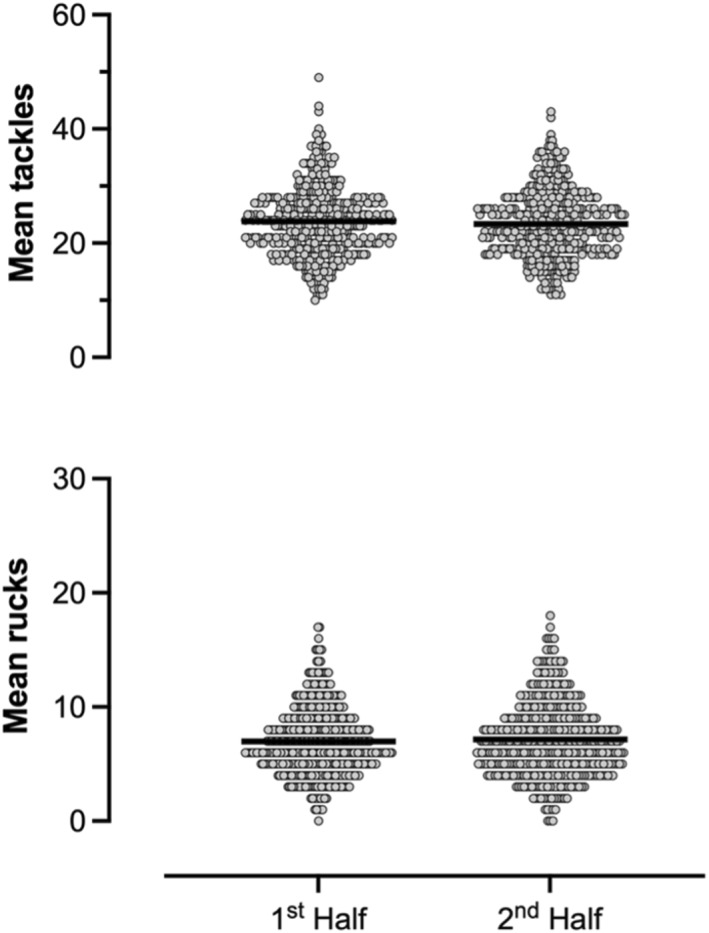
Mean frequency of tackles and rucks per half.

No differences were found between the mean tackles per match between pool and knockout stages (pool 47.5, 95% CI 46.5–48.6 vs. knockout 46.9, 95% CI 45.7–48.0). Knockout stages however had significantly fewer rucks compared to the pool stages (pool 14.8, 95% CI 14.2–15.4 vs. knockout 13.3, 95% CI 12.7–13.9, *p* < 0.001) (Figure 2). Mean tackles and mean rucks did not differ between the 1^st^ and 2^nd^ half (tackles: 1^st^ half 23.8; 95% CI 23.3–24.4 vs. 2^nd^ half 23.4, 95% CI 22.8–23.9; rucks: 1^st^ half 7.0, 95% CI 6.7–7.2 vs. 2^nd^ half 7.1, 95% CI 6.8–7.4) (Figure 3).

### 3.2 Tackle Event Variables and Frequencies

The most prevalent type of tackle was an arm tackle (mean per match 30.9, 95% CI 30.2–32.5) followed by jersey and shoulder tackles (mean per match 8.0, 95% CI 7.7–8.4 and 7.4, 95% CI 7.1–7.7, respectively) (Table 2). For each type of tackle, there were no significant differences in frequency between the competition stages and match halves. The tackler predominantly made contact with the ball‐carrier from the side (mean per match 19.6, 95% CI 19.0–20.1) followed by front‐on tackles (mean per match 13.6, 95% CI 13.1–14.1) (Table 3). The tackler also predominantly made contact with the ball‐carrier's lower half of his body (mean per match mid‐torso 23.7, 95% CI 23.3–24.3 and mean per match legs 15.8, 95% CI 15.3–16.3) (Table 4).

**TABLE 2:** Frequency of tackle events by the type of tackle. Data reported as mean (± 95%CI).

		Shoulder tackle	Arm tackle	Jersey tackle	Smother tackle	Collision tackle	Tap tackle	Lift tackle	Total
Pool									
Successful	1st half	0.8 (0.7–1.0)	2.3 (2.1–2.5)	0.3 (0.2–0.4)	0.1 (0.1–0.1)	0.03 (0.01–0.05)	0	0.004 (0–0.01)	3.5 (3.3–3.8)
	2nd half	0.8 (0.7–0.9)	2.2 (2.0–2.5)	0.3 (0.2–0.4)	0.1 (0.1–0.2)	0.03 (0.01–0.04)	0.004 (0–0.01)	0.004 (0–0.01)	3.5 (3.2–3.7)
	TOTAL	1.6 (1.4–1.8)	4.5 (4.2–4.8)	0.6 (0.5–0.7)	0.3 (0.2–0.3)	0.05 (0.03–0.08)	0.004 (0–0.01)	0.008 (0–0.02)	7.0 (6.7–7.4)
Unsuccessful	1st half	3.1 (2.8–3.3)	13.1 (12.6–13.7)	3.9 (3.6–4.2)	0.3 (0.2–0.3)	0.04 (0.01–0.06)	0.1 (0.06–0.14)	0.008 (0–0.02)	20.4 (19.7–21.1)
	2nd half	3.2 (2.9–3.4)	12.9 (12.3–13.4)	3.7 (3.4–3.9)	0.2 (0.1–0.2)	0.04 (0.01–0.06)	0.1 (0.07–0.15)	0.008 (0–0.02)	20.0 (19.2–20.7)
	TOTAL	6.3 (5.9–6.6)	26.0 (25.2–26.8)	7.5 (7.1–8.0)	0.4 (0.3–0.5)	0.08 (0.04–0.11)	0.2 (0.15–0.27)	0.017 (0–0.03)	40.5 (39.5–41.5)
Total tackle events	TOTAL	7.9 (7.4–8.3)	30.5 (29.6–31.3)	8.2 (7.7–8.6)	0.7 (0.5–0.8)	0.1 (0.1–0.2)	0.2 (0.15–0.27)	0.03 (0.01–0.04)	47.5 (46.5–48.6)
Knockout									
Successful	1st half	0.6 (0.5–0.7)	2.3 (2.1–2.5)	0.4 (0.3–0.5)	0.1 (0–0.1)	0.01 (0–0.03)	0	0	3.4 (3.1–3.7)
	2nd half	0.7 (0.5–0.8)	2.2 (1.9–2.4)	0.3 (0.2–0.4)	0.1 (0–0.1)	0.02 (0–0.05)	0	0	3.2 (2.9–3.5)
	TOTAL	1.3 (1.1–1.4)	4.5 (3.9–5.1)	0.7 (0.6–0.7)	0.1 (0.1–0.2)	0.04 (0.03–0.04)	0	0	6.6 (6.2–7.0)
Unsuccessful	1st half	2.6 (2.4–2.9)	13.4 (12.7–14.0)	3.9 (3.6–4.2)	0.2 (0.1–0.2)	0.04 (0.01–0.06)	0.1 (0.1–0.2)	0.01 (0–0.02)	20.2 (19.4–21.0)
	2nd half	3.0 (2.7–3.2)	13.5 (12.9–14.1)	3.3 (3.0–3.6)	0.1 (0.1–0.2)	0.05 (0.02–0.08)	0.1 (0.1–0.1)	0.01 (0–0.02)	20.1 (19.4–20.8)
	TOTAL	5.6 (4.9–6.4)	26.9 (23.2–30.5)	7.2 (6.2–8.2)	0.3 (0.2–0.3)	0.09 (0.07–0.10)	0.2 (0.2–0.2)	0.02 (0.02–0.02)	40.3 (39.2–41.4)
Total tackle events	TOTAL	6.9 (6.5–7.3)	31.4 (30.4–32.3)	7.9 (7.4–8.4)	0.4 (0.3–0.5)	0.1 (0.1–0.2)	0.2 (0.1–0.3)	0.02 (0–0.04)	46.9 (45.7–48.0)
Overall tackle events	TOTAL	7.4 (7.1–7.7)	30.9 (30.2–31.5)	8.0 (7.7–8.4)	0.6 (0.5–0.6)	0.13 (0.09–0.16)	0.2 (0.2–0.3)	0.02 (0.01–0.04)	47.2 (46.4–48.0)

*Note:* Pool (*n* = 239 matches) Knockout (*n* = 210 matches).

**TABLE 3:** Frequency of tackle events by the direction of contact. Data reported as mean (± 95%CI).

		Front	Side	Oblique	Behind	Total
Pool						
Successful	1st half	1.1 (1.0–1.2)	1.4 (1.3–1.6)	0.4 (0.3–0.5)	0.6 (0.5–0.7)	3.5 (3.3–3.8)
	2nd half	1.1 (1.0–1.3)	1.4 (1.2–1.6)	0.4 (0.3–0.5)	0.6 (0.5–0.7)	3.5 (3.2–3.7)
	TOTAL	2.2 (2.0–2.4)	2.8 (2.6–3.1)	0.8 (0.7–1.0)	1.2 (1.0–1.3)	7.0 (6.7–7.4)
Unsuccessful	1st half	5.9 (5.5–6.3)	8.6 (8.1–9.0)	3.0 (2.8–3.3)	3.0 (2.8–3.3)	20.4 (19.7–21.1)
	2nd half	5.8 (5.4–6.2)	8.2 (7.8–8.7)	3.0 (2.7–3.3)	3.0 (2.7–3.2)	20.0 (19.2–20.7)
	TOTAL	11.7 (11.1–12.3)	16.8 (16.1–17.5)	6.0 (5.6–6.4)	6.0 (5.7–6.3)	40.5 (39.5–41.5)
Total tackle events	TOTAL	13.9 (13.2–14.6)	19.6 (18.9–20.4)	6.9 (6.4–7.3)	7.2 (6.8–7.5)	47.5 (46.5–48.6)
Knockout						
Successful	1st half	0.8 (0.7–1.0)	1.5 (1.3–1.6)	0.4 (0.4–0.5)	0.6 (0.5–0.8)	3.4 (3.1–3.7)
	2nd half	1.1 (0.9–1.3)	1.3 (1.1–1.4)	0.4 (0.3–0.4)	0.5 (0.4–0.6)	3.2 (2.9–3.5)
	TOTAL	1.9 (1.7–2.2)	2.7 (2.5–3.0)	0.8 (0.7–0.9)	1.1 (1.0–1.3)	6.6 (6.2–7.0)
Unsuccessful	1st half	5.4 (5.0–5.8)	8.2 (7.7–8.7)	3.4 (3.1–3.7)	3.2 (2.9–3.5)	20.2 (19.4–21.0)
	2nd half	5.9 (5.5–6.3)	8.5 (8.0–9.0)	3.2 (2.9–3.5)	2.5 (2.3–2.7)	20.1 (19.4–20.8)
	TOTAL	11.3 (10.7–11.9)	16.7 (16.0–17.5)	6.6 (6.1–7.0)	5.7 (5.3–6.1)	40.3 (39.2–41.4)
Total tackle events	TOTAL	13.2 (12.5–13.9)	19.5 (18.7–20.3)	7.4 (6.9–7.9)	6.8 (6.4–7.2)	46.9 (45.7–48.0)
Overall tackle events	TOTAL	13.6 (13.1–14.1)	19.6 (19.0–20.1)	7.1 (6.8–7.4)	7.0 (6.7–7.3)	47.2 (46.4–48.0)

*Note:* Pool (*n* = 239 matches) Knockout (*n* = 210 matches).

**TABLE 4:** Frequency of tackle events by point of body contact. Data reported as mean (± 95%CI).

		Head & Neck	Shoulder	Mid‐torso	Legs	Total
Pool						
Successful	1st half	0.01 (0–0.02)	0.5 (0.4–0.6)	2.1 (1.9–2.3)	0.9 (0.8–1.1)	3.5 (3.3–3.8)
	2nd half	0.01 (0–0.03)	0.5 (0.4–0.6)	2.1 (1.9–2.3)	0.9 (0.7–1.0)	3.5 (3.2–3.7)
	TOTAL	0.02 (0–0.04)	1.0 (0.9–1.2)	4.2 (4.0–4.5)	1.8 (1.6–2.0)	7.0 (6.7–7.4)
Unsuccessful	1st half	0.2 (0.1–0.2)	3.3 (3.0–3.6)	10.0 (9.5–10.5)	7.0 (6.6–7.4)	20.4 (19.7–21.1)
	2nd half	0.2 (0.1–0.2)	3.1 (2.9–3.4)	9.7 (9.3–10.1)	7.0 (6.5–7.4)	20.0 (19.2–20.7)
	TOTAL	0.4 (0.3–0.4)	6.4 (6.0–6.9)	19.7 (19.1–20.3)	14.0 (13.3–14.6)	40.5 (39.5–41.5)
Total tackle events	TOTAL	0.4 (0.3–0.5)	7.5 (7.0–7.9)	23.9 (23.2–24.6)	15.8 (15.0–16.5)	47.5 (46.5–48.6)
Knockout						
Successful	1st half	0.03 (0–0.05)	0.5 (0–0.6)	2.0 (1.8–2.2)	0.9 (0.7–1.0)	3.4 (3.1–3.7)
	2nd half	0	0.5 (0.4–0.6)	1.9 (1.7–2.1)	0.8 (0.7–1.0)	3.2 (2.9–3.5)
	TOTAL	0.03 (0–0.05)	1.0 (0.9–1.2)	3.9 (3.6–4.1)	1.7 (1.5–1.9)	6.6 (6.1–7.0)
Unsuccessful	1st half	0.1 (0.1–0.2)	3.1 (2.8–3.3)	10.1 (9.6–10.6)	7.0 (6.6–7.4)	20.2 (19.4–21.0)
	2nd half	0.2 (0.1–0.2)	3.0 (2.8–3.3)	9.8 (9.3–10.3)	7.1 (6.7–7.5)	20.1 (19.4–20.8)
	TOTAL	0.3 (0.2–0.4)	6.1 (5.7–6.5)	19.9 (19.1–20.6)	14.1 (13.5–14.7)	40.3 (39.2–41.4)
Total tackle events	TOTAL	0.3 (0.2–0.4)	7.1 (6.7–7.5)	23.7 (22.9–24.5)	15.8 (15.1–16.4)	46.9 (45.7–48.0)
Overall tackle events	TOTAL	0.3 (0.3–0.4)	7.3 (7.0–7.6)	23.8 (23.3–24.3)	15.8 (15.3–16.3)	47.2 (46.4–48.0)

*Note:* Pool (*n* = 239 matches), Knockout (*n* = 210 matches).

### 3.3 Tackle Success Between Pool and Knockout Matches

**FIGURE 4:**
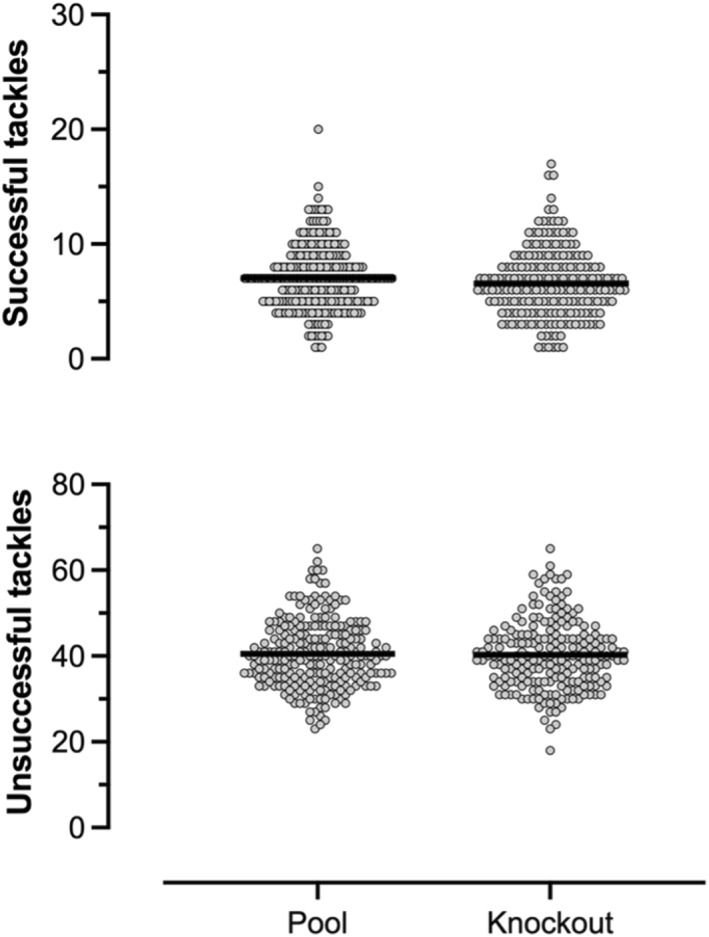
Mean frequency of tackle success across the 2018/2019 World Rugby Sevens Series stage.

Within each tournament stage, unsuccessful tackles occurred 6 times more frequently than successful tackles (successful pool 7.0, 95% CI 6.7–7.4 vs. unsuccessful pool 40.5, 95% CI 39.5–41.5; successful knockout 6.6, 95% CI 6.1–7.0 vs. unsuccessful knockout 40.3, 95% CI 39.2–41.4) (Figure 4). No differences in successful and unsuccessful tackles were found between tournament stages (successful tackles pool 7.0, 95% CI 6.7–7.4 vs. successful tackle knockout 6.6, 95% CI 6.1–7.0; unsuccessful pool 40.5, 95% CI 39.5–41.5 vs. unsuccessful knockout 40.3, 95% CI 39.2–41.4).

### 3.4 Determinants of Tackle Success

For pool stages, type of tackle, point of body contact, tackle sequence, attacker intention and match rank were significant in the model. The specific characteristics associated with tackle success for each of the significant variables are shown in Table 5A.

**TABLE 5A:** Multinomial logistic regression for tackle success in the pool stages. Data reported as relative risk ratios (RRR) and 95% confidence intervals (95% CI).

Pool					
	RRR		95% CI		*p* value
	Main	Specific	Main	Specific	Main
Successful (vs. unsuccessful) tackle					
Type of tackle (arm tackle)	0.9		0.9–1.0		< 0.001
Collision tackle		0.4		0.2–0.7**	
Jersey tackle		2.6		2.2–3.2***	
Lift tackle		0.4		0.8–2.4	
Shoulder tackle		0.7		0.6–0.9***	
Smother tackle		0.4		0.3–0.6***	
Tap tackle		5.6		0.8–41.0	
Point of body contact (mid‐torso)	0.9		0.8–0.9		< 0.001
Head and neck		4.7		1.9–11.8**	
Legs		1.8		1.6–2.1***	
Shoulder/arm		1.6		1.4–1.9***	
Direction of contact	1.0		1.0–1.1		0.274
Tackle sequence (one‐on‐one)	1.3		1.0–1.7		0.044
Sequential		1.5		1.1–2.1*	
Simultaneous		2.0		1.2–3.4*	
Number of tacklers	0.9		0.7–1.1		0.187
Attacker intention (straight)	0.8		0.8–0.8		< 0.001
Arcing run		3.1		2.4–3.8***	
Diagonal run		2.4		2.0–2.9***	
Lateral run		0.8		0.6–1.0	
Sidestep		2.2		1.8–2.7***	
Match rank (1)	0.9		0.8–1.0		0.025
2		0.8		0.7–1.0**	
3		0.8		0.7–1.0*	
Half	1.0		0.9–1.1		0.958

*Note:* Data reported as RRRs and 95% CI.

*p < 0.05; **p < 0.01; ***p < 0.001.

For knockout stages, point of body contact and attacker intention were significant in the model. The specific point of contact and attacker intention characteristics associated with tackle success during the knockout stages are shown in Table 5B.

**TABLE 5B:** Multinomial logistic regression for tackle success in the knockout stages. Data reported as relative risk ratios (RRR) and 95% confidence intervals (95% CI).

Knockout					
	RRR		95% CI		*p* value
	Main	Specific	Main	Specific	Main
Successful (vs. unsuccessful) tackle					
Type of tackle (arm tackle)	1.0		0.9–1.0		0.049
Point of body contact (mid‐torso)	0.8		0.8–0.9		< 0.001
Head and neck		2.0		0.9–4.8	
Legs		1.5		1.3–1.8***	
Shoulder/arm		1.3		1.1–1.6**	
Direction of contact	1.0		1.0–1.1		0.185
Tackle sequence	1.1		0.9–1.5		0.435
Number of tacklers	0.8		0.7–1.1		0.130
Attacker intention (straight)	0.8		0.8–0.8		< 0.001
Arcing run		4.5		3.5–5.7***	
Diagonal run		2.7		2.2–3.3***	
Lateral run		1.1		0.8–1.4	
Sidestep		2.8		2.3–3.5***	
Match rank	1.0		0.9–1.1		0.884
Half	1.1		1.0–1.2		0.242

*Note:* Data reported as RRRs and 95% CI.

*p < 0.05; **p < 0.01; ***p < 0.001.

## 4 Discussion

The average number of tackles per match remained consistent across the series (bar an increase in tackles from tournament T5 to T6), whereas the average number of rucks decreased from the first tournament to latter parts of the series. Considering the definitions used for the tackle and ruck, a plausible explanation for these findings is that the defending team develops an ability to compete in less rucks after making a tackle. In other words, as the season progresses, the defending team becomes more tactically proficient and avoids contesting for the ball on the ground after tackling the ball‐carrier. In Sevens, the number of players competing in the ruck has been shown to be a key performance indicator with attacking teams at risk of losing ball possession and conceding points when they commit more than one attacker to contest the ruck (Hendricks et al. 2020; Ross et al. 2016). The performance role of fewer rucks in Sevens was also highlighted by the difference in ruck frequencies between the pool and knockout stages. While every ruck is an opportunity for the defending team to contest and regain possession of the ball, in Sevens, contesting fewer rucks may seem tactically superior. Using Markov chain analysis on a sample of 117 matches to identify variations in pattern behaviour between the different competition stages, Barkell, Pope, et al. (2017) showed scoring from turnovers was a key differentiator between winning and losing teams during the pool and knockout stages. Winning teams consistently score from turnovers throughout the pool and knockout stages, whereas losing teams rarely scored from turnovers (Barkell, Pope, et al. 2017). Considering the Barkell, Pope, et al. (2017) findings in conjunction with ours, it may not just be a case of simply contesting fewer rucks. Rather, the decision as to which rucks to contest or not may be a key performance determinant. From a defencive training perspective, coaches should emphasise better post‐tackle decision‐making as to whether or not to contest the ruck.

In both the pool and knockout stages, the most frequently occurring type of tackle was the arm tackle. In the pool stages, the arm tackle was also associated with a higher likelihood of tackle success compared to other tackle types, bar jersey tackles. While the frequency and higher likelihood of tackle success for arm tackles in Sevens is not surprising considering the available space to players, this finding highlights a tackle demand unique to Sevens. In rugby union, the most frequently occurring type of tackle with the highest likelihood of tackle success is the active shoulder tackle (Hendricks et al. 2014 2018; Till et al. 2023). This difference as to which tackle type is associated with tackle performance emphasises the need for Sevens specific tackle training and coaching (Hendricks et al. 2018a; Behardien et al. 2024). Hendricks et al. (2020) also found arm tackles decreased the likelihood of offloads (compared to jersey tackles); however, the authors also found smother tackles to be more beneficial than arm tackles in reducing offloads. For point of contact on the ball‐carrier, Hendricks et al. (2020) also found that contacting the ball‐carrier at the legs (compared to contacting the mid‐torso) increased the probability of an offload, whereas contacting the arms/shoulders reduced the probability of an unsuccessful outcome—which is inconsistent with our findings where contacting the ball‐carrier at the legs and arms/shoulders increased the likelihood of a successful tackle. When comparing the two studies, it is worth noting that, in addition to the difference in sample size (4799 tackles vs. 21226), the definition for tackle success also differed. The current study recorded a successful tackle when the tackler prevented the ball‐carrier and ball from progressing towards his try‐line and did not concede a penalty. In other words, when the tackler prevented an offload, a tackle break or did not commit an infringement. In contrast, the aforementioned findings reported for Hendricks et al. (2020) focused on offloads only (i.e., excluded tackle breaks and possession lost). Nonetheless, both studies provide insights into the technical determinants of tackle success, which is an understudied area in Sevens (Burger, Lambert, and Hendricks 2020). These studies can be used to address the growing need to develop evidence‐based training programmes specifically for Sevens (Schuster et al. 2017; Hendricks et al. 2018 2018a; Behardien et al. 2024).

No differences in tackle characteristics were found between competition stages and between match halves. Two previous studies comparing match halves have also found no differences in contact frequency between match halves (Suarez‐Arrones et al. 2014; Peeters et al. 2019). In team sports, one of the main reasons for studying differences in match periods is to understand the influence of fatigue (Suarez‐Arrones et al. 2016). Considering the short duration of Sevens matches, the lack of differences in tackle characteristics between match halves suggests that match fatigue may not be as influential on overall tackle performance. Having said that, the ability to repeatedly contest the tackle is highly reliant on players' technical capacities (Hendricks et al. 2020a), and studies have shown how physical and mental fatigue can reduce players tackling technique (Davidow et al., 2020 2023). The current study did not analyse tackle technique; therefore, further studies are required to understand the effects of fatigue on technique and tackle performance and injury risk in Sevens.

For competition stages, although Barkell, Pope, et al. (2017) showed differences in game patterns between pool and knockout stages, for example, in the knockout stages, winning teams were more likely to regain possession after a kick, our findings show that tackle characteristics remain consistent. The consistency in tackle characteristics between competition stages and between match halves provides further insights into the unique tackle demands of Sevens. A practical implication of these findings is that coaches and practitioners should train players' robustness to repeatedly perform a range of tackles.

## 5 Strength and Limitations

A major strength of the current study is the analyses of an entire World Seven Series, which equated to 21226 tackle contact events and 6345 rucks events. Furthermore, the study used both video analyses approaches in one study that is describing the frequency of the tackle and ruck in different competition stages and match periods (‘the what’) and the magnitude for success of key determinants for these contact events (‘the how’). Using both video analyses approaches in one study however limited the number of tackle determinants that could be analysed. In other words, because of the total amount of tackles, only five key variables in contact were analysed. In a sample of 4799 tackle events, Hendricks et al. (2020) was able to study 10 contact, post‐contact, and match context determinants, along with more detailed tackle outcomes. Furthermore, the authors were able to study determinants of success during the ruck as well. Ultimately, the amount of contact events and the detail around each event are dependent on project objectives, timelines and resources.

## 6 Conclusions

In conclusion, tackle frequencies per match remained consistent across the series and between the different competition stages and match halves. Ruck frequencies on the other hand decreased from the first tournament to latter parts of the series, and fewer rucks were observed in the knockout stage of the tournaments. Based on these findings, contesting fewer rucks after a tackle may be a tactical consideration from a defencive perspective. With that said, which rucks to contest or not may also play a role, therefore coaches should emphasise better post‐tackle decision‐making. In both the pool and knockout stages, the most frequently occurring type of tackle was the arm tackle. In the pool stages, the arm and jersey tackle were also associated with a higher likelihood of tackle success compared to other tackle types. The frequency and higher likelihood of tackle success for arm tackles in Sevens highlights a unique demand of Sevens, which strengthens the argument for Sevens specific tackle training and coaching. The consistency in tackle characteristics between competition stages and between match halves provides further insights into the unique tackle demands of Sevens, with players requiring robustness to repeatedly perform a range of tackles.

## Conflicts of Interest

SH is the social media editor and an associate editor for the European Journal of Sport Sciences.
